# The intersectional genetics landscape for humans

**DOI:** 10.1093/gigascience/giaa083

**Published:** 2020-08-06

**Authors:** Andre Macedo, Alisson M Gontijo

**Affiliations:** Chronic Diseases Research Center, NOVA Medical School, Faculdade de Ciências Médicas, Universidade Nova de Lisboa, Rua do Instituto Bacteriológico 5, 1150–190, Lisbon, Portugal; Chronic Diseases Research Center, NOVA Medical School, Faculdade de Ciências Médicas, Universidade Nova de Lisboa, Rua do Instituto Bacteriológico 5, 1150–190, Lisbon, Portugal

**Keywords:** combinatorial genetics, gene regulation, cell targeting, cell classifier, enhancers, promoters

## Abstract

**Background:**

The human body is made up of hundreds—perhaps thousands—of cell types and states, most of which are currently inaccessible genetically. Intersectional genetic approaches can increase the number of genetically accessible cells, but the scope and safety of these approaches have not been systematically assessed. A typical intersectional method acts like an “AND" logic gate by converting the input of 2 or more active, yet unspecific, regulatory elements (REs) into a single cell type specific synthetic output.

**Results:**

Here, we systematically assessed the intersectional genetics landscape of the human genome using a subset of cells from a large RE usage atlas (Functional ANnoTation Of the Mammalian genome 5 consortium, FANTOM5) obtained by cap analysis of gene expression sequencing (CAGE-seq). We developed the heuristics and algorithms to retrieve and quality-rank “AND" gate intersections. Of the 154 primary cell types surveyed, >90% can be distinguished from each other with as few as 3 to 4 active REs, with quantifiable safety and robustness. We call these minimal intersections of active REs with cell-type diagnostic potential “versatile entry codes" (VEnCodes). Each of the 158 cancer cell types surveyed could also be distinguished from the healthy primary cell types with small VEnCodes, most of which were robust to intra- and interindividual variation. Methods for the cross-validation of CAGE-seq–derived VEnCodes and for the extraction of VEnCodes from pooled single-cell sequencing data are also presented.

**Conclusions:**

Our work provides a systematic view of the intersectional genetics landscape in humans and demonstrates the potential of these approaches for future gene delivery technologies.

## Introduction

The exact number of different cell types that makes up the body of a human adult is yet to be defined, but is expected to be in the order of several hundred, or perhaps thousands of different cell types [1, 2]. Major efforts have recently been launched to attempt to catalogue and molecularly describe every cell type in different tissues of the human body [3–8]. The number and the complexity of cell types increase further when one considers that cells exist in different states, not only when a cell divides or undergoes successive differentiation steps during normal developmental processes, but also when a cell becomes infected or cancerous, or specifically responds to physical or chemical stimuli [1, 2, 5].

A major challenge in biology and biomedicine has been to genetically identify and deliver genetically encoded messages to a specific cellular type and/or state within complex organisms. Most gene delivery systems are limited by the technology available to distinguish the desired cellular types and/or states between themselves prior to gene delivery; most technologies rely primarily on cell-surface markers for selectivity [9, 10]. These markers are seldom cell-specific, and this lack of specificity inevitably leads to DNA delivery to unwanted cells. This can have negative consequences, such as introducing undesired artifacts in research studies or side effects in gene therapy–based interventions. Additionally, the usage of sporadically defined cell surface markers for cellular targeting restricts both the ability to systematize the generation of cell-specific gene delivery vectors and to scale this system up for any cell type or state in any organism.

An alternative to these “pre-DNA delivery” selectivity procedures is to use cell type– and cell state–unspecific viral or non-viral DNA delivery systems [11, 12], and work out the cell specificity post-delivery by exploring the unique genetic properties of the target cell. The transcriptional program of any given cell reflects, at the most basic level, a unique combination of binary on/off states of the regulatory elements (REs) present in the genome. REs can be used multiple times by different cells either at different anatomical sites, different time points of life history, or during disease or environmental responses [3, 4, 13–17]. Therefore, while the activity of a single, carefully chosen RE could theoretically provide sufficient specificity to identify a particular cell type and/or state post–DNA delivery in some cases, it is unlikely to provide the required specificity to distinguish most cell types and/or states between themselves [13, 14].

**Figure 1:**
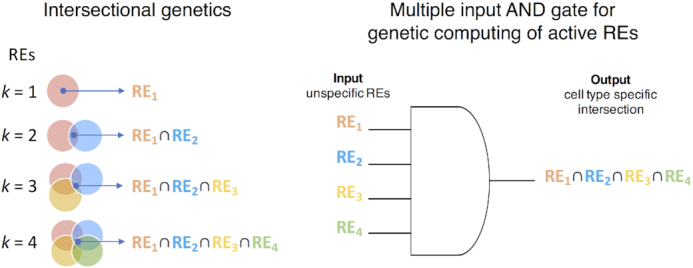
Intersectional genetics. Scheme of the intersectional genetics approach to obtain cell type–specific drivers by restricting expression to the cells where 2 or more REs with broader activity overlap (intersect). REs are the inputs that will pass through a typical “AND" logic gate and give a single, genetically defined output in the cells where the RE activities intersect.

Aware of this fact, developmental biologists studying model organisms have devised intersectional genetic methods to increase the target cell specificity of gene drivers by exploring the anatomical overlap between expression patterns driven by 2 independent REs [14, 18–23]. Similarly, molecular and synthetic biologists have engineered systems that use Boolean logic to sense different cell states in bacteria and yeast [24, 25]. In many of these synthetic computational systems, the REs are the inputs which will pass through a typical “AND" gate and give a single genetically defined output (Fig. 1). Similar systems have been applied to mammalian cells, where they are able to distinguish between different cancer cell types or detect cancer cells arising from normal cells *in vitro* [26–28]. Despite being successful, the full potential of this type of intersectional approach has never been evaluated or applied systematically to generate drivers for every cell type in a body, and even less so for a complex organism like a human, which lacks thoroughly developmentally characterized gene drivers.

Here, we hypothesized that the majority of cell types and/or cell states in human could be distinguished post–DNA delivery using multiple input “AND" gates (intersectional methods of active REs; Fig. 1), and that the intersecting inputs could be obtained, quality-ranked, and cross-validated using currently publicly available RE usage databases.

## Materials and Methods

### Data preparation and normalization

To quantify how cellular specificity scales with the number of intersecting active REs (*k*), we developed algorithms and scripts using the Python language to analyze genome-wide data on promoter and enhancer usage for hundreds of primary human cell types obtained by the FANTOM5 consortium [3, 4, 29]. Briefly, the FANTOM5 data consists of curated subsets of transcriptional start site “peaks” determined by capped analyses of gene expression sequencing (CAGE-seq). The height of each CAGE-seq peak provides quantitative information in normalized tags per million (TPM) values, which is interpreted as being directly proportional to the activity of the promoter or enhancer that it represents.

Before analyzing the FANTOM5 data, we manually curated the FANTOM5 human cell type database, consisting of 184 distinct cell types from multiple donors (giving a total of 562 data sets), by selecting for healthy primary cells and removing cell treatments/infections and cells obtained from cancer samples (Supplementary Fig. S1). We also attempted to remove data sets that were less likely to represent single cell types. Examples of the samples removed during curation are: data sets from cells infected with *Salmonella* or *Candida albicans*, data sets for cells labeled “whole blood,” and data sets from mesenchymal precursor cells obtained from cancer samples. Some data sets were merged into a single cell type category; for example, “CD8+ T Cells (pluriselect)” and “CD8+ T Cells” and “Melanocyte dark” and “Melanocyte light” were treated as 2 single cell type categories. This curation resulted in a list of 154 distinct primary cell types from multiple donors, giving a total of 537 samples and averaging ∼3.5 samples (donors) per cell type (range, 2–6). Supplementary Table S1 contains the list of curated cell types used in this study, as well as all of the excluded and merged categories.

The total number of possible RE combinations for a target cell type is *C(r, k) = (r!/(k! (r—k)! )*, where *r* stands for the number of REs of the database (e.g., 201,802 promoters in FANTOM5), and *k* stands for the number of REs chosen to combine. For *k* = 4, this gives 6.9 × 10^19^ possible combinations. To ask whether any combination is specific for the target cell type, however, we need to ask whether the *k* combined elements are all active in the given cell type and at least 1 of the *k* elements is inactive in each of the other cell types in the database. If the *k* elements could be binarized into active (TRUE) and inactive (FALSE) categories, this question can be asked using Boolean logic gate functions such as (in conjunctive normal form): ((*^1^k_1_*AND *^1^k_2_*AND…*^1^k_n_*) AND (NOT *^2^k_1_*OR NOT*^2^k_2_*OR NOT…*. ^2^k_n_*) AND (NOT *^3^k_1_*OR NOT*^3^k_2_…*OR NOT*^3^k_n_*)… AND (NOT *^n^k_1_*OR NOT*^n^k_2_…*OR NOT*^n^k_n_*)), where ^c^*^{1^*$\to $*^n^*^}^*k_{1_*$\to $*_n_*_}_ represents the status of the RE element *k* in cell type *c* (where the target cell type is 1). The truth table for this function has 2^(c*k)^ rows, which for 154 cell types and *k* = 4 gives 2.7 × 10^185^ rows. Saturating the search for all possible combinations for any given cell type and testing them by a brute-force algorithm requires polynomial time complexity *O*( [c*r]*^k^*).

To increase the tractability of this problem, the size of the database for a given cell type can be reduced for each search using heuristic methods. For instance, REs that are inactive in the target cell or active in the target cell and also active in most other non-target tissues (e.g., REs of housekeeping genes) are not helpful for the purpose of making cell type–specific intersectional gene drivers.

**Figure 2:**
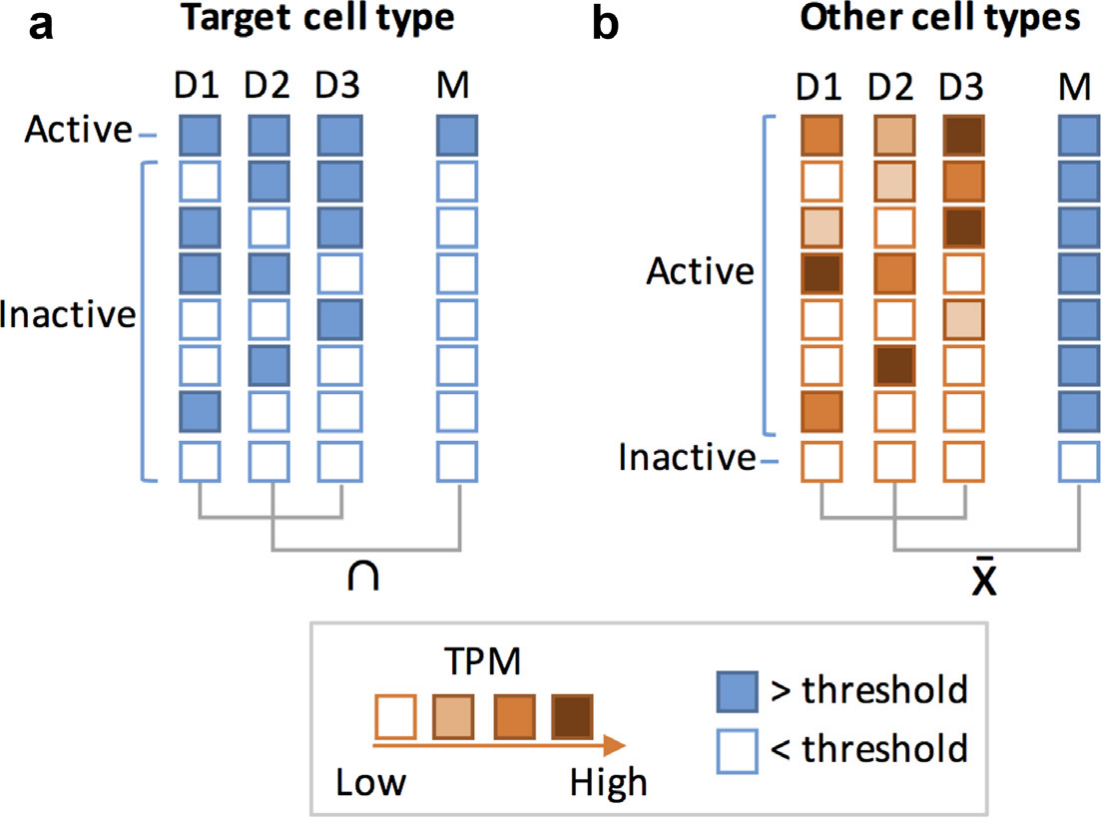
Conservative criteria for RE activity. Different conservative criteria for RE activity were applied to (**a**) target and (**b**) non-target cells (“other cell types”). Each row represents a possible RE activity scenario. Each box represents the activity of the RE per donor (D) or the collapsed intersection or average (M), according to the color key. REs from target cells were considered active if the intersection of all cell donors was above a TPM threshold (blue squares). REs from other cell types were considered active if the average raw TPM (M) of all donors was above the threshold.

Hence, to increase the likelihood of finding fruitful intersections and to reduce the database complexity and computing time, we applied several filters on the database to select for sparsely active REs. The first step is to define RE activity thresholds. We decided to be conservative and apply different activity thresholds for the target cell type and for the non-target cell types. This would increase the chances that the selected REs are truly active in the target cell type and inactive in the non-target cell type. To reduce the database size and concentrate on potentially active REs in the target cell type, we created subsets of data for each target cell type where we retained only the REs that were consistently potentially “ON" (>0 TPM) in all donors for that cell type (Fig. 2a). We next collapsed the data from multiple donors of the non-target cell types to a single non-target cell type data point by averaging the expression of the multiple donors (Fig. 2b). This reduces the database complexity by a factor of ∼3.5.

To select for sparsely active REs, we studied the RE activity landscape by testing the following thresholds for RE activity in the target cell type: 0.5, 1, and 2 TPM. The higher the RE activity threshold, the more stringent the RE selection is. For inactivity, we tested 0, 0.01, and 0.1 TPM in non-target cells. By applying these thresholds, we transform the continuous CAGE-seq peak data into binary data sets.

**Figure 3:**
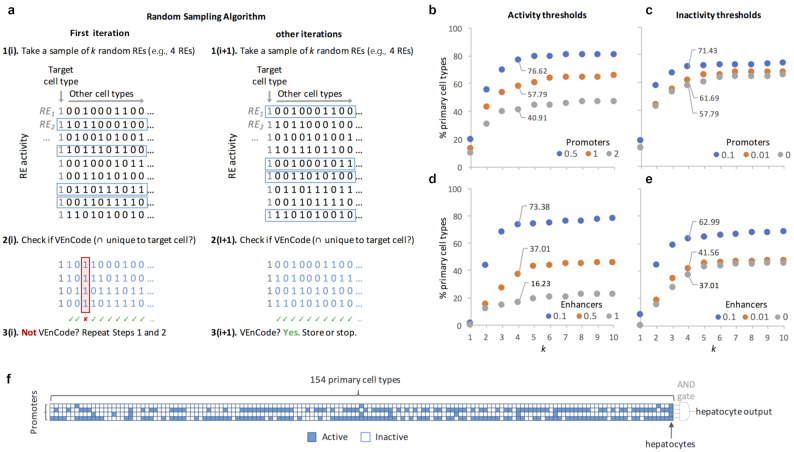
Random sampling method to find intersecting active REs (versatile entry codes [VEnCode]). (**a**) Rationale for the sampling method. First, *k* REs are randomly selected from the set of REs that are active (“1”) in the target cell type. Inactive REs are depicted as “0.” Then, we ask whether, if at least 1 sampled RE is inactive in each other cell type in the data set. If yes, these *k* REs satisfy VEnCode criteria or the target cell type (e.g., the *k* REs must intersect exclusively in the target cell). If not, we repeat steps 1 and 2. If the *k* REs satisfy VEnCode criteria in the first or second iteration (i+1), then the *k* RE selection is counted as a VEnCode and is stored. Probing the intersection genetics landscape for (**b, c**) promoter and (**d, e**) enhancer data sets using the sampling method. Plotted are the percentages of cell types found to have at least 1 VEnCode per *k* and different (b, d) activity and (c, e) inactivity TPM thresholds. For the activity panels, the inactivity threshold was fixed at 0 for both promoters and enhancers. For the inactivity panels, the activity thresholds were fixed at 0.5 and 0.1 for promoters and enhancers, respectively. (**f**) Visual representation of a VEnCode for hepatocytes. Binary heat map where each column represents 1 of the 154 primary human cell types and each row 1 RE from the promoter data set. Blue signifies an active RE and white an inactive RE.

We then wrote a program that randomly samples the filtered RE landscape by choosing a combination of *k* “active” REs for a target cell type and asking whether this combination is exclusive to the target cell type, as compared to the other cell types of the database. We call this the sampling method (Fig. 3a).

To further reduce computing time, the algorithm first selects for sparsely active REs by removing all REs that are active in more than X% of the cell types. This removes broadly expressed REs. We start with X = 90%, but decrement 5 units (i.e., 85%) each time there are not enough REs left in the data set after the filter (e.g., *n* of REs < *k*). We ran this sampling program up to *n* = 1,000 times for a *k* range of 1 to 10, and calculated the percentage of cells for which at least 1 exclusive combination for the target cell type was found. This percentage served as an indicator of the cellular specificity of combinations of *k* active REs.

## Results

### Random sampling of intersecting active REs

Using promoter data from the sub-panel of 154 primary human cell types, we find that cellular specificity of *k* intersecting REs increases logarithmically from 10–20% for *k =* 1 up to a plateau of 40–80% starting at *k* = 5, depending on the activity threshold (0.5–2 TPM, with a fixed inactivity threshold at 0 TPM; Fig. 3b). The 0.5 TPM activity threshold gave the highest selectivity. Relaxing the inactivity thresholds from 0 to 0.1 TPM (with a fixed activity threshold at 1 TPM) increased the percentage of cells that could be detected by 10–15%, depending on the *k* used, again reaching a plateau at around *k = 5* (Fig. 3c). A similar scenario was observed using enhancer data, albeit the activity threshold that gave the highest selectivity was lower (0.1 TPM) than for promoters, likely reflecting the generally lower TPM values of the enhancer subset (Fig. 3d). Relaxing the inactivity thresholds up to 0.1 (with a fixed activity threshold of 0.5 TPM) did not improve the cell selectivity (Fig. 3e). These results suggest that combinations of just a handful of active REs could provide substantial cellular resolution in humans. As predicted, the usage of a single input (*k =* 1) has a very limited potential to detect cell types or cell states. Moreover, even though a 2-input “AND" gate greatly increases the number of detectable cell types, it is unlikely to provide the breadth required to be applicable for a technique aimed at detecting most cell types and/or states in the human body. Finally, at least for this data set and methodology used, our results suggest that our ability to sort cell types based on active RE intersections plateaus between 4–6 REs.

Safety is also a concern when considering possible human applications of RE activity-based methods, such as unwanted leakage (noisy or unpredicted RE activity) in cell-targeted therapies. Using high *k* values would be beneficial in this sense, because for each extra *k* there is an extra safety layer to account for false negatives when compared to *k = 1*. Namely, the probability *p* of leakage decreases exponentially by *p^k^*. By applying the simple RE selection criteria described above (with activity thresholds of 0.5 and 0.1 TPM for promoters and enhancers and a strict inactivity threshold of 0 TPM for both), the usage of a 4-input “AND" gate (*k =* 4 combination of promoters and/or enhancers), which can theoretically add as many as 3 safety layers against false negatives when compared to *k =* 1, is able to discern ∼77% and ∼73% of human cell types using promoters and enhancers, respectively (Fig. 3b and d), suggesting that it is a good compromise between technical feasibility (i.e., generating biological systems that use 4 REs and translating the activity of the gene products regulated by these REs into a single genetic readout) and the breadth of cell types that can be detected. These multiple-input “AND" gates can also be seen as the minimal intersection of co-activated REs that is diagnostic of a given cell type or state within a given complex mixture of cells in a culture dish, in a tissue biopsy sample, or in the human body. We call these intersecting active REs “versatile entry codes" (VEnCodes; Fig. 3f).

### Heuristic selection of intersecting active REs

**Figure 4:**
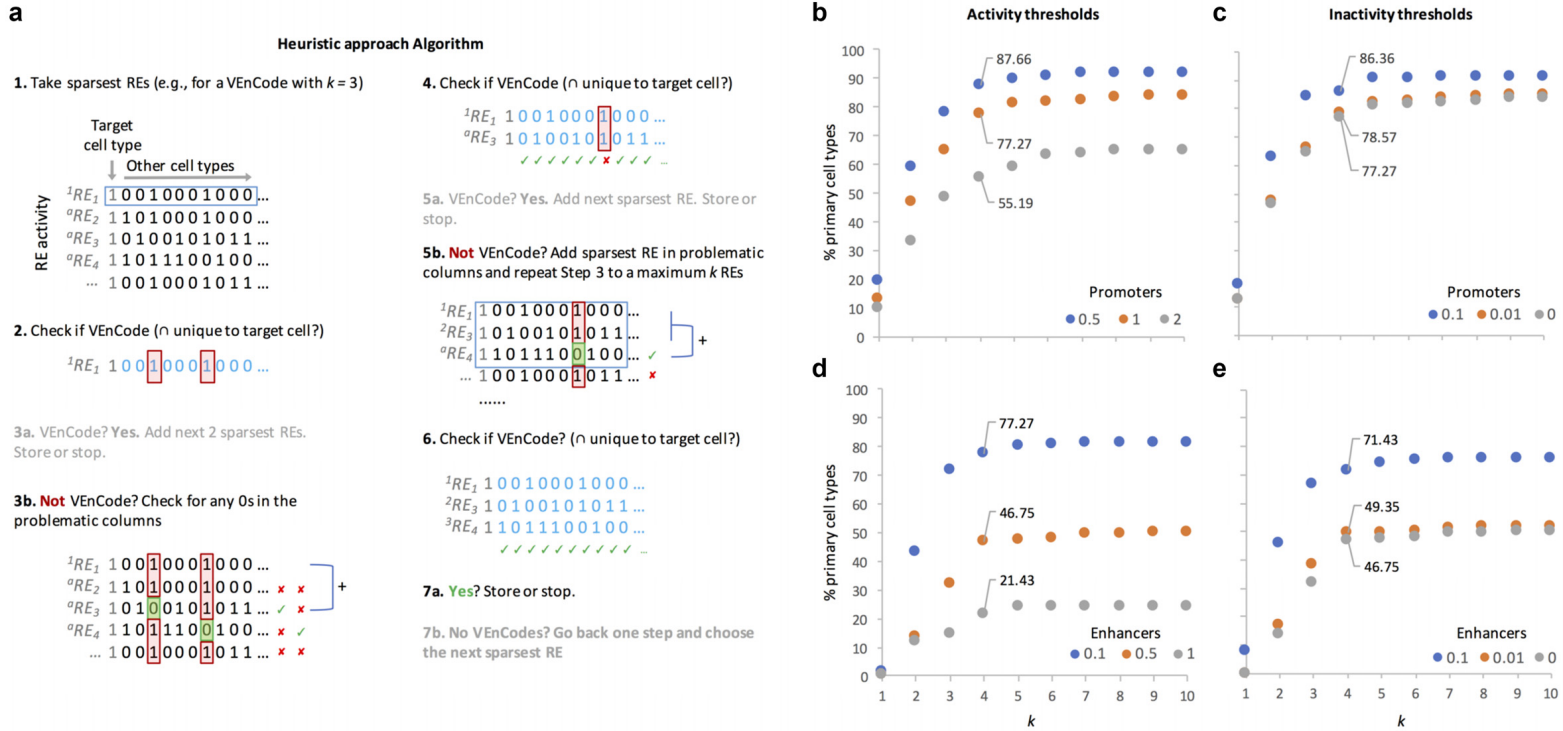
Heuristic method to find intersecting, active REs (VEnCodes). (**a**) Rationale for the heuristic method. An example is given for a VEnCode with *k* = 3. This algorithm follows a greedy strategy where at each node of the decision tree it makes the locally optimal choice. First, it sorts the REs in the data set by sparseness, then it takes the sparsest RE (first-level node) and asks whether it is inactive in all non-target cell types. If yes, this RE is cell-type specific, and the next *k*-1 sparsest REs can be added to increase safety. If not, it finds out in which cell types this RE is active and searches the data set for a new RE that is inactive in those problematic cell types. If successful, the intersection between these 2 REs will be specific for the target cell type. In case no RE matches the query, it reorders the REs by sparseness, this time calculating sparseness only at the “problematic” cell types. It then chooses the sparsest RE as the second-level node and repeats the procedure as described for the first node, increasing node depth until a VEnCode is found. Node depth is always ≤*k* and the algorithm tests several nodes at each level before it gives up. In the example given, there was no need to reorder by sparseness, as there was a satisfactory VEnCode. Probing the intersection genetics landscape for (**b, c**) promoter and (**d, e**) enhancer data sets using the heuristic method. Plotted are the percentages of cell types found to have at least 1 VEnCode per k and different (b, d) activity and (c, e) inactivity TPM thresholds. For the activity panels, the inactivity threshold was fixed at 0 for both promoters and enhancers. For the inactivity panels, the activity thresholds were fixed at 0.5 and 0.1 for promoters and enhancers, respectively.

The random sampling method still falls short of probing the enormous landscape of possible VEnCodes. We thus attempted a heuristic approach to probe the VEnCode landscape. We used the same binarization criteria as in the sampling method but removed the filter for sparsely active REs that would retain REs that were active in a percentage of the cell types assayed. This was done since the effectiveness of this approach is not affected by a large data set of less sparsely active REs. REs occupy the rows of the database and can be represented as *^a{1^*$\to $*^k^*^}^RE*_b_*, where “*a”* represents the position of the RE in the VEnCode (e.g., for a VEnCode with *k* intersections, *a* will go from 1 to *k*) and *b* represents the row number in the RE list. We then applied a greedy algorithm that considers the sparseness of expression (Fig. 4a). In brief, the REs are first sorted by expression sparseness, and the sparsest RE (RE_1_) is chosen as a first-order position (hereafter, “node”) ^1^RE_1_. All cell type columns in which the^1^RE_1_ activity is 0 are then culled from the database, and all remaining ^>^^1^RE_>1_ are resorted in ascending fashion according to the number of cell types they have that share co-activity with ^1^RE_1_. Then, ^1^RE_1_ is tested in combination with the next RE (^2^RE_2_) to verify whether it satisfies criteria as a VEnCode (i.e., whether the intersection between the active REs ^1^RE_1_ ∩ ^2^RE_2_ occurs exclusively in the target cell samples). It follows that for each *k =* 2 combination that satisfies the VEnCode criteria, all further *k > 2* combinations that use these 2 REs will satisfy the criteria for the VEnCode. If no *k = 2* combination satisfies the VEnCode criteria, the algorithm creates secondary nodes and reiterates the pattern described above. To increase the coverage of the landscape, each multiple node test is performed with the 3 nearest neighbors by order of sparseness. If no *k* = 3 combination satisfies VEnCode criteria, the algorithm creates tertiary nodes, and so on. We call this approach the heuristic approach.

Applying the heuristic approach to search for VEnCodes using similar threshold conditions as used for the sampling method above, we obtained cell-specific combinations of *k* promoters and enhancers for ∼90% and ∼80% of the cell types, respectively (Fig. 4b–e). More importantly, this method shifts leftwards the plateau for the maximum number of cell types detected, so that we are now able to retrieve specific combinations for a larger percentage of cell types even at lower *k* numbers. For instance, at *k =* 4 we retrieve ∼88% and ∼77% of cell types when using promoters and enhancers, respectively. To test whether the cells that could not be retrieved shared a common developmental origin or similar expression profile, we performed single-linkage clustering using the CAGE-seq TPM expression data for the 154 primary cell types and highlighted the cell types with 0 VEnCodes retrieved using the heuristic method and *k* = 4 (Supplementary Figures S2 and S3). We found that cells without VEnCodes frequently clustered together in groups of 2 or more cell types (such as the cluster of 5 CD4+ cell types). Moreover, some of the distances between nodes are very small, especially for enhancer expression profiles, explaining why some “isolated” cell types could not be distinguished.

**Figure 5:**
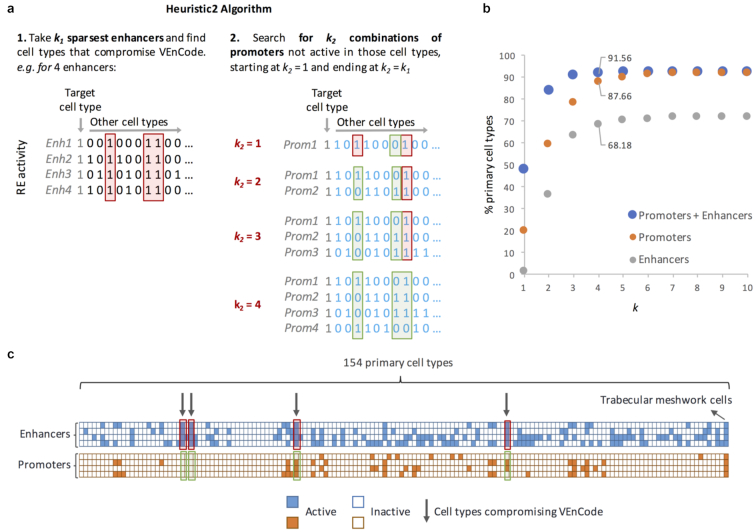
Heuristic2 method to find intersecting, active REs (VEnCodes). (**a**) Rationale for the Heuristic2 method. This algorithm combines the efficiency of the heuristic method with the extra flexibility of using both enhancers and promoters to target a cell type. First, it finds the *k* sparsest enhancers (*k_1_*) that are active for the target cell type and asks whether they are a VEnCode. If they are not, it focuses on the “problematic” cell types in which the enhancers are active and, using the approach described in Fig. 4, asks whether there is any combination of promoters (*k_2_*) that are not active in those cell types. If so, then the intersection of the enhancer and promoter activities is specific to the target cell type. (**b**) Probing the intersection genetics landscape using the heuristic2 method. Plotted are the percentages of cell types found to have at least 1 VEnCode per *k* using promoter (orange circles), enhancer (gray circles), or promoter + enhancer (blue circles) data. (**c**) Visual representation of a VEnCode obtained using the Heuristic2 method for trabecular meshwork cells. Binary heat map where each column represents 1 of the 154 primary human cell types and each row 1 RE from the enhancer (blue boxes) and promoter (orange boxes) data sets. Red boxes and arrows depict the cell-type data that are preventing the interception of enhancers from being a VEnCode for the trabecular meshwork cells. Green boxes highlight the promoter expression data in those problematic cell types.

To try to find VEnCodes for the cell types where they could not be retrieved using either the sampling or the heuristic method, we combined enhancer (*k_1_)* and promoter (*k_2_*) data in a method we called the “heuristic2” approach (Fig. 5a). This method increases cellular resolution to ∼85% of cell types for *k = 2* (combinations of 2 *k_1_* enhancers and 2 *k_2_* promoters) and > 90% of cell types for *k* = 4 (combinations of 4 *k_1_* enhancers and 4 *k_2_* promoters; Fig. 5b), allowing the generation of VEnCodes for difficult cell types that could not be resolved using promoter or enhancer data alone (Fig. 5c). Even though none of our methods saturate the RE activity intersection landscape, these results consistently indicate that combinations of just a few active REs could provide substantial cell type resolution in human.

### Measuring VEnCode robustness

**Figure 6:**
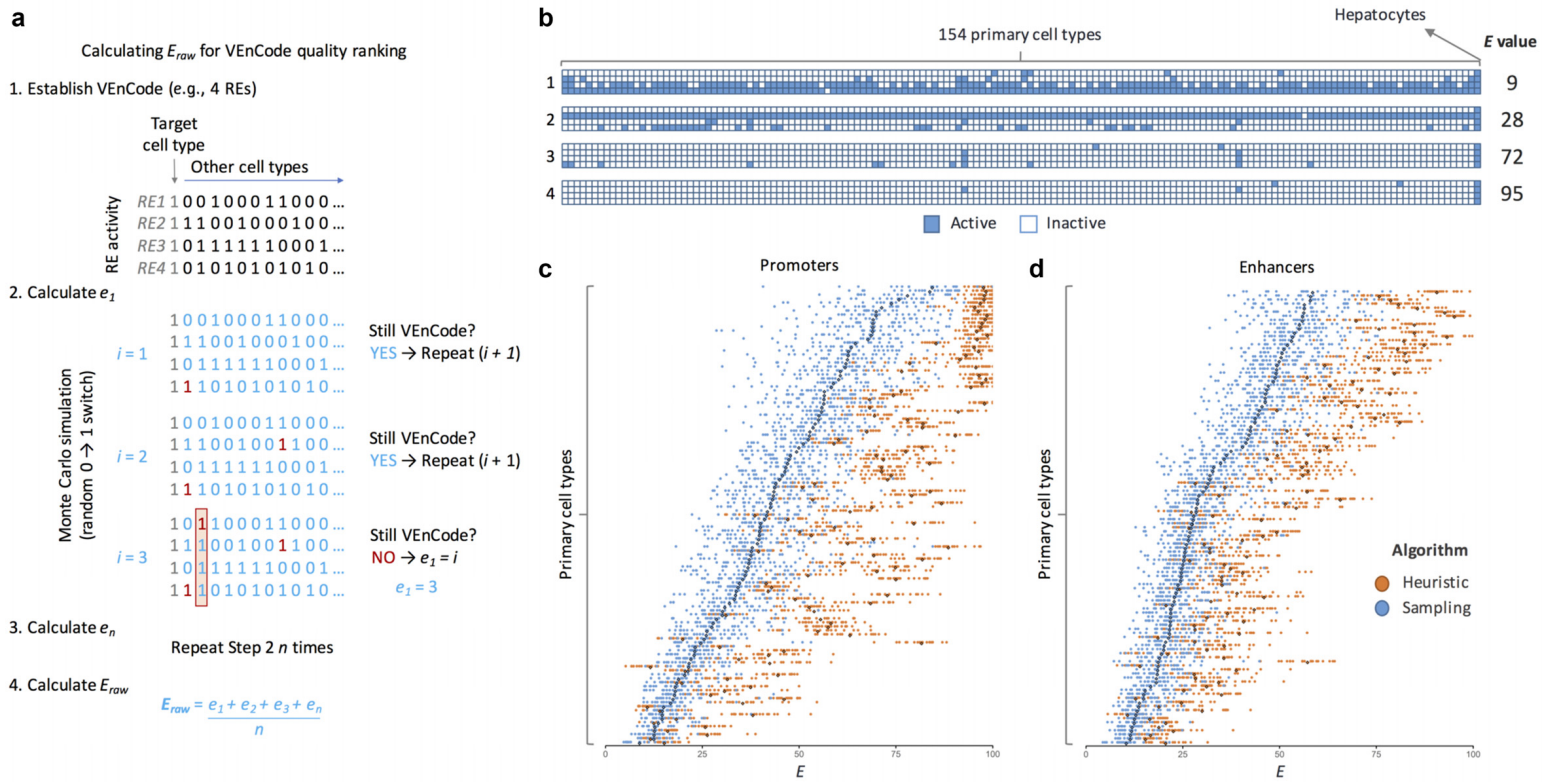
Method for ranking VEnCode intraindividual robustness. (**a**) Outline of the method to calculate the *E* value of a VEnCode. *E*_raw_ is calculated by taking a VEnCode (1) and accounting for possible false negatives in the data by turning inactive REs into active ones (2). To this end, the algorithm performs random 0-to-1 changes in the data set, 1 at a time, and then checks whether the VEnCode condition is still satisfied. It reiterates *e_1_* times until the VEnCode condition is no longer satisfied. It then repeats the simulation *n* times (3) and returns *E_raw_* by calculating the average of all *e* values obtained (4). *E_raw_* is then normalized according to the formula described in Supplementary Figure S2 and Supplementary Table S2 to obtain *E*. (**b**) Visual representation of 4 (1–4) hepatocyte VEnCodes obtained using different algorithms and promoter data. Binary heat map where each column represents 1 of the 154 primary human cell types and each row 1 RE from the promoter data (blue boxes). The *E* value of each VEnCode is depicted on the right. (**c, d**) The effect on *E* values of using sampling (blue) or heuristic (orange) methods to obtain VEnCodes. The heuristic method increases the average *E* for most cell types for (c) promoter and (d) enhancer data. The *y* axis represents different cell types ordered by increasing *E* obtained by the sampling method. Each dot is a VEnCode (*n* = 5–20 per primary cell type). Darker diamonds represent the mean.

Next, we asked whether we could devise algorithms to rank a VEnCode according to its quality and robustness. A *k =* 4 VEnCode assumes, based on the available RE usage data, that the 4 chosen REs are never active together in any cell type and/or state except in the desired target cell type and/or state. Clearly, there could be many instances when this premise is false, so that the VEnCode falls apart. For instance, the VEnCode is compromised if the VEnCode is also able to detect a cell type which is not included in the database used or if false negatives are a prevalent artifact of the databases used to devise VEnCodes (e.g., a given RE is labeled as inactive in our database but in reality is active or, for any reason, unstably fluctuates between active and inactive states). To attempt to quantify these problems, we carried out Monte Carlo simulations of false negative results by randomly activating REs and recalculating whether or not the VEnCode continued being selective for our target cell type after each simulation (Fig. 6a). We scored how many false negatives, on average (for *n* simulations), are required until the VEnCode falls apart. This gives the quality value *E_raw_* for each VEnCode. *E_raw_* varies as a function of *k* comprising the VEnCode and the number of cell types *c* in the database. The higher the *k*, the higher the*E_raw_*, attesting to the fact that intersections are more robust to technical errors and biological noise. To make *E* comparable between different conditions, we normalize *E_raw_* according to a reference best-case scenario *E_best(c, k)_* value, which was obtained by the Monte Carlo simulations performed as described above, yet for the best-case scenario for a VEnCode: where all *k* REs are inactive in the non-target cell types. Hence, normalized *E* = 100*E_raw_/E_best(c, k)_ (Supplementary Fig. S4 and Supplementary Table S2). The idea is that *E* is directly proportional to the intraindividual robustness of a given VEnCode towards a cell type (Fig. 6b).

To understand how *E* scales with cell type identity, we used the sampling method to obtain an unbiased set of VEnCodes using *k =*4 promoters. From the 114/154 cell types for which we retrieved 5–20 VEnCodes in *n =*10,000 samplings, we obtained *E* values varying between 6 and 99 (Fig. 6c and Supplementary Fig. S5A). The *E* quality index varied substantially between cell types. For instance, “Fibroblast—Mammary” cells only allow the generation of VEnCodes with small *E* values (between 5 and 17), while hepatocytes allow the generation of high-quality VEnCodes with large *E* values (between 62 and 91). To test whether the heuristic method improved the VEnCode quality, we calculated *E* from a subset of 5–20 promoter VEnCodes obtained from 131 and 114 cell types using the heuristic and sampling methods, respectively (Fig. 6c and Supplementary Fig. S5B). As expected, the heuristic method statistically significantly improved the VEnCode quality by an average of 21.1 units (range, 6–57) above random sampling for 88.5% of cell types (100/113; *P <* 0.0005; Bonferroni-corrected, unpaired Student's *t-*tests; Fig. 6c). Similar results were obtained for enhancer VEnCodes: there was an average improvement of 14.1 units (range, 4–40) over random sampling for 83% of cell types (93/112; *P <* 0.00005; Bonferroni-corrected, unpaired Student's *t*-tests; Fig. 6d and Supplementary Fig. S5C-D). We conclude that the heuristic method not only finds VEnCodes for a larger amount of cell types, but also generates higher-quality VEnCodes.

### VEnCode interindividual robustness

**Figure 7:**
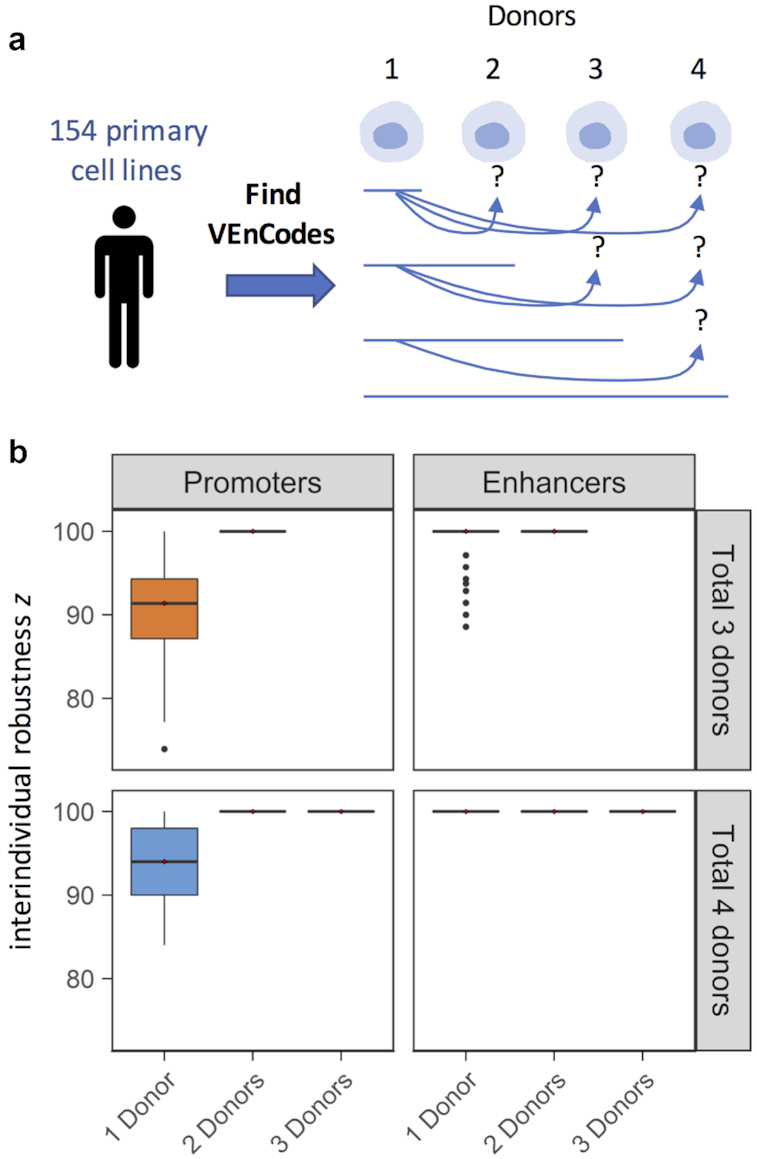
VEnCode inter-individual robustness. (**a**) Rationale for the estimation of VEnCode interindividual robustness. VEnCodes that are generated based on data from 1 or more donors are tested as VEnCodes on data from other donors. The percentage of VEnCodes that satisfy VEnCode criteria for the other donors is *z*. (**b**) Box plots representing *z* are obtained from various primary cell types based on promoter and enhancer data. Subsets of primary cells for which 3 (top panels, orange) and 4 (bottom panels, blue) donors were tested. *z* is saturated at 100% for all cell types tested when VEnCodes are determined using data from 2 cell donors.

An ideal VEnCode retains its specificity towards the target cell type across multiple individuals of a population. For this, the VEnCode must be robust to interindividual variation on cell-specific RE usage patterns. Interindividual variation could arise either due to technical variation introduced during the determination of active and inactive REs for a given cell type in a given individual or as a true biological variation in RE usage for that cell type between individuals. The likelihood of relying on false positive calls to generate VEnCodes should be inversely proportional to the number of individuals surveyed for RE usage in the target cell type. To verify this, we estimated interindividual robustness *z* of VEnCodes by calculating the percentage of VEnCodes generated from a subset of cell type donors that retained the VEnCode ability to satisfy all other donors of that cell type whose data were not used to generate the initial VEnCodes (Fig. 7a). Our results show that despite some variability between interindividual robustness across different cell types, on average, their VEnCodes (*k =* 4) are robust (Fig. 7b). Namely, when promoter usage data from 1 and 2 donors are used, the *z* values increase, on average, by ∼9.4%, from 90.6 to 100% (*P <* 0.00001; Wilcoxon test for a subset of 66 cell types with 3 donors with 0 < n < 71 VEnCodes generated by the sampling method in all conditions; Fig. 7b). Similar results were found for a subset of cell types (*n* = 9) with 4 donors, where 2 donors were sufficient to saturate *z* (Fig. 7b). Enhancer data from a single donor seems to carry even more predictability for other donors than promoter data, as *z* is only 1.4% lower than 100% on average when data from 1 donor is used instead of 2 (*P* = 0.00096; Wilcoxon test for a subset of 67 cell types with 3 donors; Fig. 7b). When the subset of 6 cell types with enhancer data from 4 donors was analyzed, data from a single donor was sufficient to saturate *z* (Fig. 7b). These results suggest that using data from more than 1 donor is most helpful for promoter data, where it can help significantly increase VEnCode interindividual robustness and, hence, increase the likelihood that a VEnCode will be specific for the target cell in different individuals.

Even though there is no correlation between the average VEnCode quality *E* for a cell type and the cell type's interindividual robustness *z* (Supplementary Fig. S6), consistent with the facts that an interindividually robust VEnCode need not be of high *E* quality and that a VEnCode with a high *E* score is not necessarily the best VEnCode for multiple individuals, the optimal scenario would be to determine VEnCodes from a large cohort of donors of a cell type and then choose the VEnCodes with highest *E* scores from this subset. With this in mind, we calculated the 5 best VEnCodes using the heuristic2 method, with *k* ranging from 1 to 4 for a list of primary cell types with at least 3 donors (Supplementary Data S1). This list can serve as a starting point to explore other properties of VEnCodes and to perform cross-validation experiments using independent techniques, similar to what we report further below.

### VEnCodes for alternative cell states: cancer

**Figure 8:**
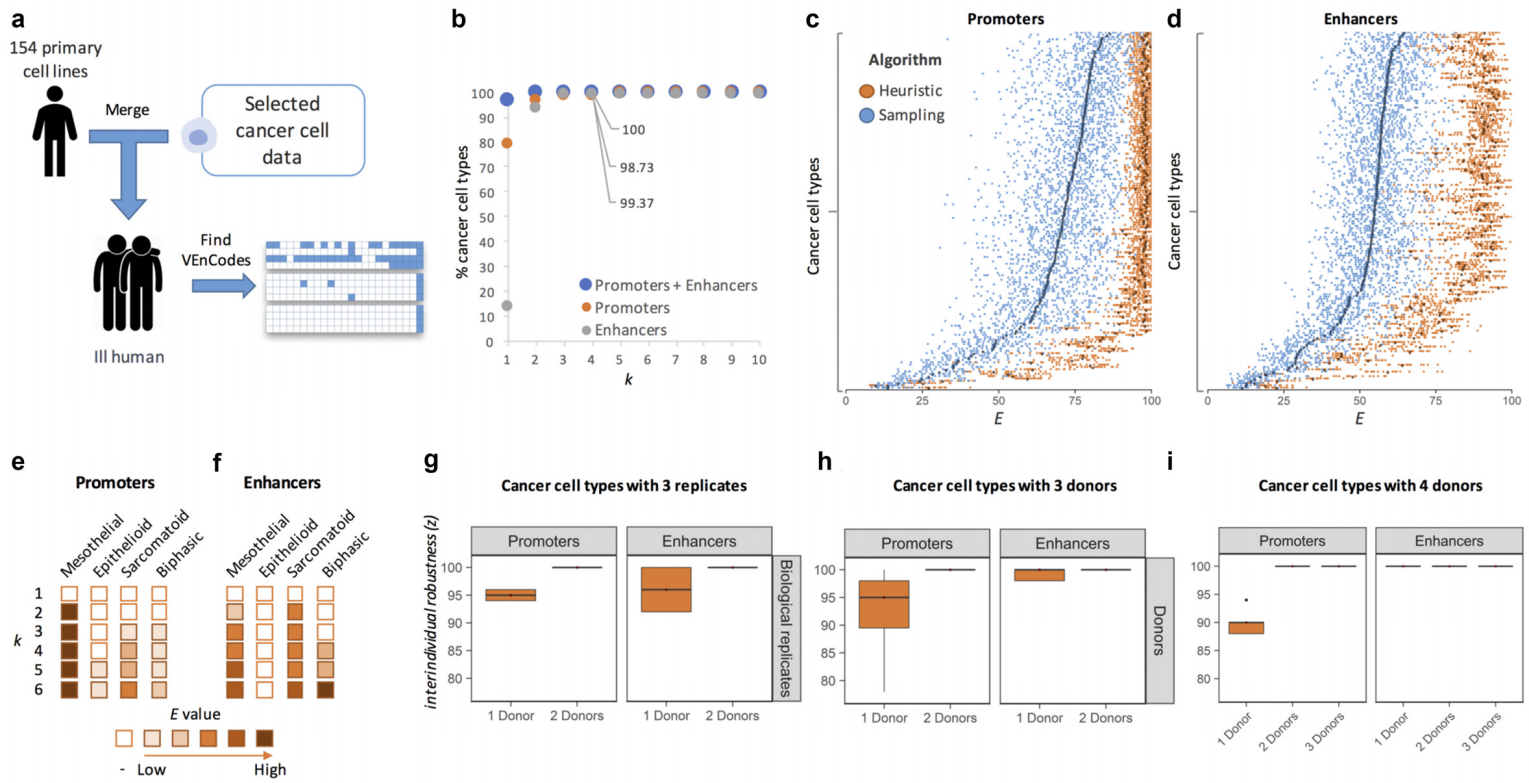
VEnCodes for cancer cell types. (**a**) Strategy for simulating a cancer patient in silico. (**b**) Probing the intersection genetics landscape for cancer cell types using the Heuristic2 method. Plotted are the percentages of cell types found to have at least 1 VEnCode per *k* using promoter (orange circles), enhancer (gray circles), or promoter + enhancer (blue circles) data. (**c, d**) The effect on *E* values of using sampling (blue) or heuristic (orange) methods to obtain VEnCodes for cancer cell types. The heuristic method increases the average *E* for most cell types for (c) promoter and (d) enhancer data. The*y* axis represents different cancer cell types ordered by increasing the*E* obtained by the sampling method. Each dot is a VEnCode (*n* = 5–20 per primary cell type). Darker diamonds represent the mean. (**e, f**) Case study of mesothelioma cancer cells stratified into epithelioid, sarcomatoid, and biphasic subtypes. Primary mesothelial cells are shown in the left column for reference. Rows depict increasing *k*. Boxes are filled if at least 1 VEnCode is found using *k* REs. If a VEnCode is found, the box is colored according to the binned average *E* value of the VEnCodes found (*n* = 1–20). (**g–i**) Box plots representing interindividual robustness *z* values obtained from all cancer cell types with (g, h) 3 or (**i**) 4 donors based on promoter (left panels) and enhancer (right panels) data. (g) Subsets of cancer cells for which biological replicates were available (i.e., repeated assays with the same cancer cell line). (h, i) Subsets of cancer cell types for which independent cell lines were analyzed. *z* is saturated at 100% for all cell types tested when VEnCodes are determined using data from 2 cell donors.

The FANTOM5 database contains RE usage data for 274 cancer cell line samples [3, 4, 29], which can be merged into 158 cancer cell types (Supplementary Table S3). If VEnCodes could be determined for cancer cell types, they could be used in different cell targeting methods, such as to improve cell selectivity in gene therapy directed towards cancer cells. To verify whether VEnCodes could be determined for cancer cell types, we created *in silico* models for diseased patients carrying 1 cancer cell type each by adding the cancer cell type data to the 154 primary cell type database (Fig. 8a). While there are many caveats and sources of additional noise with this strategy, such as cell line heterogeneity, long-term cell culture artifacts, cell donor gender, and the incompleteness of the cell type and state database used, to cite just a few, it already serves the purpose of submitting the cancer cell types to the same stringent criteria as if they were a new primary cell type. Furthermore, the availability of data from cancer cell types obtained from multiple donors provides the possibility to test for interindividual robustness of the cancer cell VEnCodes.

Exploring the RE landscape of cancer cell types, we again noticed that VEnCodes are readily obtained even for smaller *k* values (Fig. 8b), except for enhancers, where only ∼14% of cancer cell types had a specific enhancer (*k* = 1; Fig. 8b). At *k =* 4, ∼99% of cancer cell types surveyed could be distinguished using the heuristic method for promoters or enhancers (Fig. 8b). This goes up to 100% using the heuristic2 method with a *k = 3* (Fig. 8b). Cancer cell type VEnCodes are generally of very high quality, as shown by their large *E* values (Fig. 8c and d). Using the heuristic method increases the *E* values, similar to what we observed in primary cell lines (Fig. 8c and d).

A caveat of the *in silico* cancer patient model is that not all cells of origin of some cancer cell types are present in the primary cell database. This is the case for small cell lung carcinoma (SCLC), which is thought to originate from neuroendocrine cells of the lung [30]. Certainly, an expansion of the primary cell database is warranted, and it would help generate safer and more robust VEnCodes.

To study this issue more carefully, we looked at mesothelioma, for which the assumed primary cell of origin, the mesothelial cell, is available in the current database. We first stratified the mesothelioma cell types into 3 cytological classes according to Cellosaurus [31]: epithelioid (*n* = 7: ACC-MESO-1, ACC-MESO-4, Mero-14, Mero-41, Mero-82, Mero-95, NCI-H226, and No36; epithelial-like stellate cells), sarcomatoid (*n* = 3: NCI-H2052, NCI-H28, and ONE58), and biphasic (*n* = 5: Mero-25, Mero-48a, Mero-83, Mero-84, NCI-H2452). We then asked how difficult it was to generate robust VEnCodes for these mesothelioma types (Fig. 8e and f). We find that while VEnCodes can be readily generated for primary mesothelial cells with *k =* 2, larger *k* values are required to generate VEnCodes for mesothelioma cells. VEnCodes were found for all mesothelioma subtypes, except for epithelioid mesothelioma cells, which could only be identified when promoter data were used, and even then they were of poor quality (*E =*∼7). In general, the VEnCode intraindividual robustness *E* increased with a higher *k*, again attesting to the potential safety value of using more intersections (Fig. 8e and f).

As many cancer cell types are characterized by a level of heterogeneity, we were expecting less interindividual robustness in cancer cells relative to primary cell types. We thus applied the sampling method to calculate the interindividual robustness *z* of cancer cell types. We found that cancer cell type VEnCodes (*k =* 4) determined either from promoter or enhancer usage data have very high interindividual robustness *z*, which is already saturated when data from 2 donors are used (Fig. 8g–i). These results show that small RE usage signatures can reproducibly define dozens of cancer cell types. The level of interindividual robustness is similar to that of technical replicates (Fig. 8g, compare top and bottom panels). Even genetically hypervariable cancer cell types, such as SCLC cells [32], for which data from 4 cell lines were available, also gave 100% *z* values when data from 2 donors were used (Fig. 8i). We conclude that highly robust and safe cancer cell VEnCodes can be obtained using CAGE-seq data.

### VEnCode cross-validation

Having shown that a publicly available RE usage database based on CAGE-seq data can be used to generate and quality-rank VEnCodes for hundreds of primary and cancer cell types, we next asked whether these CAGE-seq–based VEnCodes could be cross-validated using other publicly available comprehensive RE usage data sets. There are 2 types of cross-validation that would be desirable: first, to show that all the REs used in the VEnCodes for a given cell type are indeed active in that cell type; and second, to show that the combination of enhancers is exclusively active in that cell type.

To cross-validate CAGE-seq VEnCodes for a specific cell type using RE activity estimated by other methods in the same cell type, we searched the literature for suitable studies and found 18 candidate cell types that could be used for cross-validation (Table S4): 3 “healthy” cell types (human-induced pluripotent stem cells [hiPSCs] and 2 primary cell types), and 15 cancer cell types/lines. Whereas FANTOM5 data on hiPSCs were not included in our curated primary cell database, the fact that hiPSCs and human embryonic stem cells share nearly identical molecular profiles and pluripotency properties [33–35] allows the usage of a high-quality functional enhancer data set generated for human embryonic stem cells [36] for VEnCode cross-validation. The methods for RE activity estimation in the retrieved studies varied between chromatin immunoprecipitation–based methods, DNA accessibility–based methods (Formaldehyde-Assisted Isolation of Regulatory Elements, FAIRE; DNase I hypersensitive site mapping, DHS; Assay for Transposase-Accessible Chromatin using sequencing, ATAC-seq), enhancer function methods (enhancer RNA detection methods, eRNA; self-transcribing active regulatory region sequencing, STARR-seq), and combinations of these methods (Supplementary Table S4). We downloaded raw data (Browser Extensible Data, BED; FASTA; Comma-Separated Values, CSV; BroadPeak; or Tab-Separated Values, TSV, files) from the studies and parsed the data to retrieve compatible genomic locations of “active” or potentially active enhancers (for DNAse accessibility-dependent techniques).

**Figure 9:**
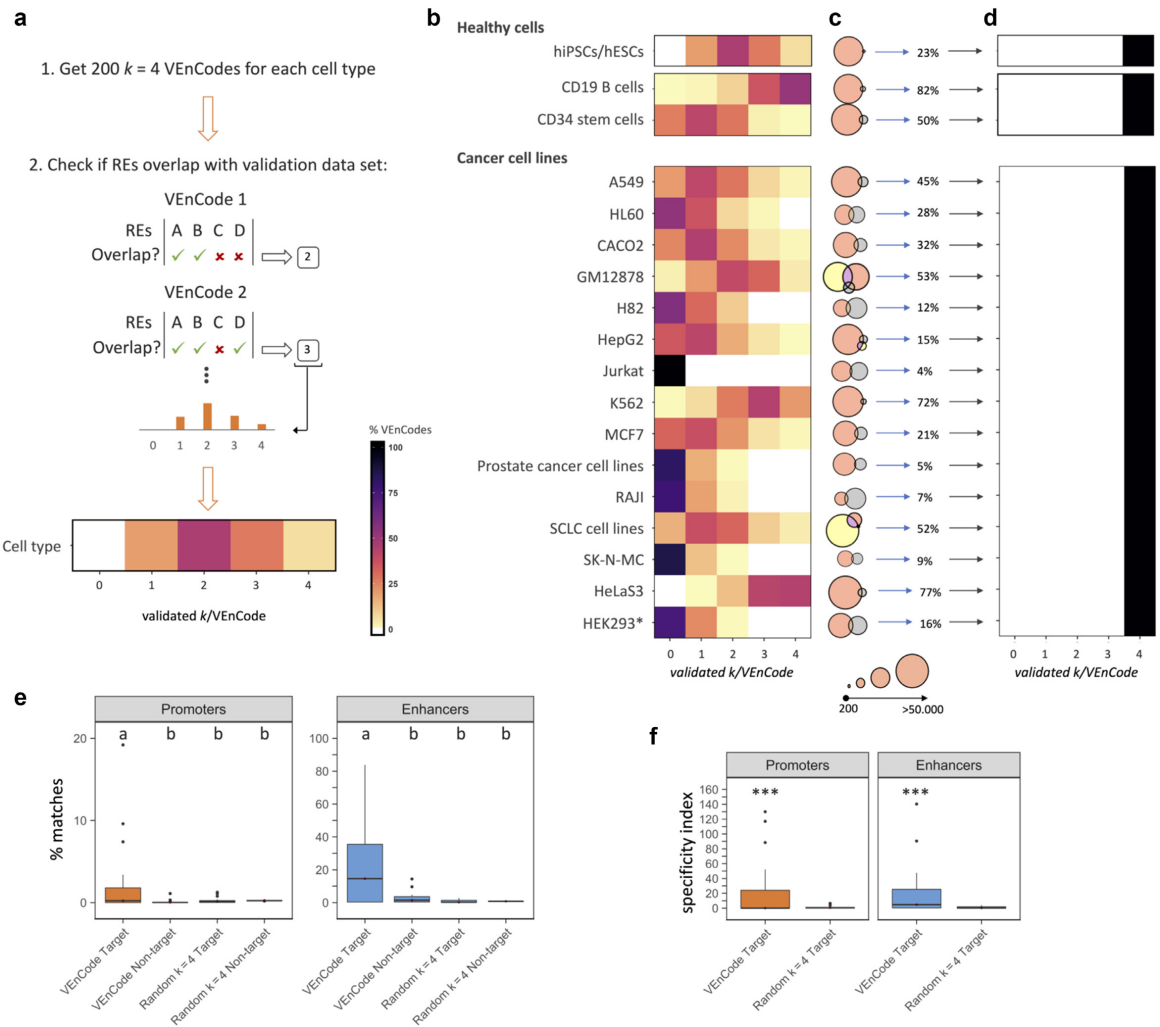
Cross-validation of CAGE-seq–determined VEnCodes. (**a**) Strategy for cross-validation of CAGE-seq–determined VEnCodes using external databases. (**b**) Heat maps depicting the distribution of cross-validated VEnCodes, for *k =* 4 enhancers, according to the number of validated *k/*VEnCode for each cell type. The databases used for cross-validation of each cell type and the full description of the cell types are provided in Supplementary Table S4. (**c**) Venn diagrams depicting the percentage of active enhancer calls determined in this study using the FANTOM5 database CAGE-seq data (gray circles; notice that they are sometimes too small to see in the figure) that overlap with the external cross-validation databases (peach and yellow). (**d**) Heat maps depicting the distribution of cross-validated VEnCodes, for *k =* 4 enhancers, according to the number of validated *k/*VEnCode for each cell type using exclusively CAGE-seq enhancers that are present in the external databases. *HEK293 cells originate from fetal kidney tissue, but they were placed in this group as they are derived from adenovirus-transformation and have a complex karyotype. (**e**) Box plots depict the percent matches between CAGE-seq–based VEnCodes and DNase-seq regions. Both VEnCodes and random combinations of *k* = 4 REs were retrieved from the CAGE-seq data set and their activity was assessed in the DNase-seq data set for both similar (target) and unrelated (non-target) cell types. Different letters represent conditions that are statistically significantly different (ANOVA followed by Tukey HSD; P < 0.01). (**f**) Specificity of CAGE-seq–derived combinations of REs (VEnCodes or random *k =* 4 combinations). Specificity was calculated as the percentage of target cell hits/average percentage of non-target cell hits. *** represents *P*< 0.001.

To cross-validate the CAGE-seq–determined VEnCodes, we generated up to 200 VEnCodes (with *k* = 4) for each of the 18 cell types and determined the fraction of *k* per VEnCodes that were considered active in the external database (Fig. 9a). Any degree of overlap (>0 nucleotides) between the CAGE-seq RE coordinates and the external database active RE coordinates was considered positive for validation. Cross-validation results varied between cell types and studies. Fully validated *k* = 4 VEnCodes, for instance, were found for 11/18 (61.1%) cells, while partially validated (≥2/4) VEnCodes were found in 17/18 (94.4%) cell types (Fig. 9b). The low validation for some cell types could be due to many factors, such as cell identity and the nature of the method used to determine RE activity. Indeed, the likelihood of VEnCode cross-validation correlated significantly with the percentage overlap of active RE calls between CAGE-seq and the external method (*r* = 0.90; *P <* 0.00001; Supplementary Fig. S7). This is not unexpected, as the readouts used to determine enhancer activity in the different studies vary in specificity and sensitivity. Regardless of these limitations, our results indicate that for a majority of cell types, CAGE-seq VEnCodes can be cross-validated using RE usage data sets independently determined by other methods. To bypass the limitation of low overlap between enhancer activity calls in different databases, we first cross-validated enhancers and then determined VEnCodes using the curated primary cell CAGE-seq data (Fig. 9c). With this approach, cross-validated VEnCodes were obtained for all 18 cell types (Fig. 9d), and these VEnCodes could be further quality-ranked according to their *E* value (Supplementary Fig. S8 and Supplementary Data S2). Hence, high-quality VEnCodes can be found using exclusively the subset of CAGE-seq REs that are cross-validated against publicly available RE usage data sets.

To perform the second type of cross-validation—to address whether combinations of enhancers are exclusively active in a given cell type—we used the ENCODE DNAse-seq database [15]. While there is a correlation between CAGE- and DNAse-seq calls, the readouts of the techniques are not the same. CAGE-seq measures the transcriptional activity of promoters and enhancers, whereas DNAse-seq measures the DNA accessibility of any locus to a DNAse enzyme. DNAse-seq can be positive in active or inactive REs and even in other functional DNA elements, such as origins of replication. Despite these potential caveats, we expected to detect nonrandom associations while scanning the DNAse-seq database using our CAGE-seq VEnCodes. To test this, we curated the ENCODE DNAse-seq database and found 27 cells that matched the CAGE-seq human primary cell types and for which we could generate VEnCodes for promoters and enhancers (Supplementary Fig. S9). We then generated up to 500 *k =* 4 CAGE-seq–based VEnCodes per cell type and quantified cell type specificity by asking how many of these CAGE-seq VEnCodes matched each of the cell types of the DNAse-seq database. These results were statistically compared to those obtained using randomly chosen *k =* 4 CAGE-seq–determined RE combinations. Results showed that CAGE-seq VEnCodes matched their target cell more frequently than random combinations when either promoters or enhancers were used (*P <* 0.05; paired Student's *t*-test; Fig. 9e) and matched non-target cells less frequently than randomly chosen *k =* 4 RE combinations for promoters, but not enhancers (*P <* 0.05; Fig. 9e). A specificity index (percentage target cell hits/average percentage non-target cell hits) showed that VEnCodes from CAGE-seq REs were more specific than randomly chosen *k =* 4 combinations or CAGE-seq REs (Fig. 9f). These results further support the cross-validation of VEnCodes described above. As regards the presence of non-target cell type hits, it is also important to consider that some cell types, in particular endothelial cells and fibroblasts, generated matches to other endothelial and fibroblast cells collected from other anatomical sites (Supplementary Fig. S9). Considering the limitations of the CAGE-seq and DNAse-seq comparisons, we conclude that VEnCodes are expected to be highly specific.

### VEnCodes using single-cell sequencing data

Genome-wide RE activity profiles have traditionally been obtained using large cell populations, as in the case of the FANTOM5 CAGE-seq data studied herein. While the advantage of these bulk preparations is the increased depth and resolution of the RE activity predictions obtained, a clear disadvantage is the loss of single-cell resolution. This is most evident in situations where RNA is prepared from complex mixtures of cells, such as healthy tissues samples and cancer biopsies. Single-cell strategies that can both resolve cell heterogeneity and infer RE activity have been developed, but they still provide a relatively shallow (discontinuous) and noisy view of RE activity per cell [37–43]. These properties limit the usefulness of single-cell strategies for VEnCode determination, which requires very stringent RE activity criteria, especially for negative RE activity calls. Single-cell CAGE-seq (C1 CAGE), for instance, recovers, per cell, on average, ∼15, ∼10, ∼5, and <5% of bulk CAGE-seq–determined enhancers expressed at 10–100, 5–10, 1–5 and < 1 TPM, respectively [43]. Considering that the thresholds for enhancer inactivity and activity in our study are 0 and > 0.5 TPM, this suggests that the rate of false positives in C1 CAGE–determined VEnCodes would be high.

Some of the limitations described above can be partially circumvented using strategies that consolidate single-cell RE activity profiles by pooling a sufficiently large number of single cells after taxonomic cell clustering [44–52]. We thus hypothesized that pooled C1 CAGE seqeuence data could retrieve enough enhancers to enter the VEnCode determination pipeline.

**Figure 10:**
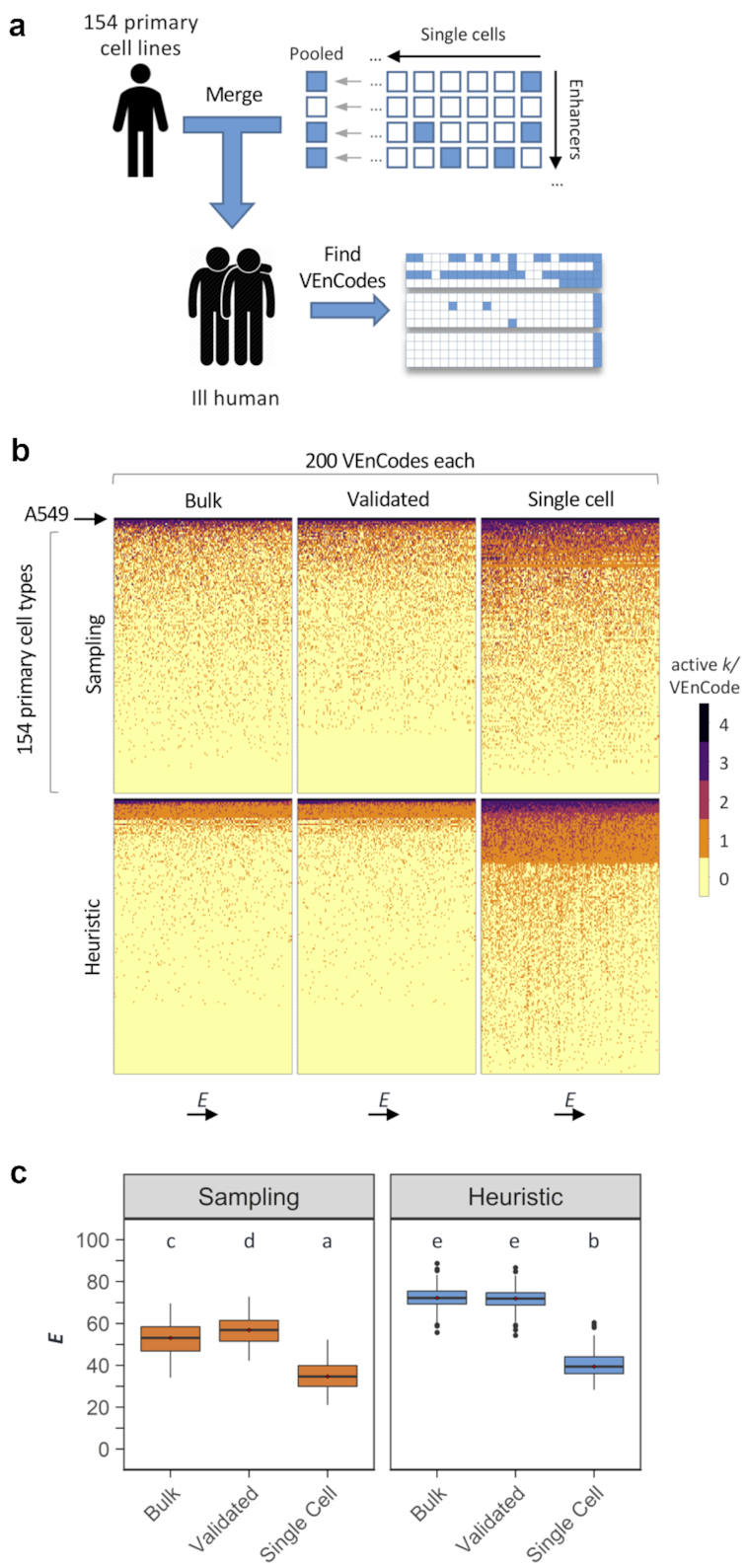
VEnCodes from single cell–derived RE usage data. (**a**) Strategy for integrating single cell–derived RE usage data into the VEnCode-generation pipeline. Single-cell data, such as enhancer usage patterns obtained from C1 CAGE-seq, are pooled and then integrated into the curated database of the bulk CAGE-seq enhancer panel. (**b**) Heat maps depicting the *E* quality of 200 *k =* 4 enhancer VEnCodes obtained by the sampling or heuristic method from single-cell (35 pooled “T0” cells from C1 CAGE-seq [43]), bulk CAGE-seq (FANTOM5), or bulk-validated CAGE-seq data (as described in Fig. 9) for A549 cells. Columns represent 200 VEnCodes ranked according to *E* value; rows represent cell types, ranked according to average ( [active *k*)]*/*VEnCode). (**c**) Box plots representing the quantification of the *E* value data reported in section b. Different letters represent conditions that are statistically significantly different (ANOVA performed with all 6 conditions, followed by Tukey HSD; *P < 0*.01).

To test this, we obtained pooled enhancer activity prediction data from a subset of untreated adenocarcinomic human alveolar basal epithelial cell lines (“T0” [untreated] A549 cells; *n* = 35 cells; Supplementary Table S5) [43]. From this set of cells, we retrieved 540 unique enhancers that were considered “ON” (Supplementary Table S6). All other non-retrieved enhancers were considered “OFF.” This data was then processed as described above for bulk CAGE-sequenced cancer cells (Fig. 10a). Results showed that VEnCodes could be successfully retrieved from enhancer activity profiles obtained from pooled C1 CAGE data (Fig. 10b). In general, C1 CAGE–based VEnCodes obtained by the sampling method were 34.1 and 38.3% worse in quality than bulk and bulk-validated CAGE-seq–determined VEnCodes, respectively (average ± SD *E* values of 35.0 ± 6.7, 53.1 ± 7.5, and 56.7 ± 6.90 for C1 CAGE, bulk, and bulk-validated CAGE-seq enhancers, respectively; *P <* 0.01 for each respective comparison; Tukey HSD (honestly significant difference) post hoc test; Fig. 10b and c). A similar pattern was observed for VEnCodes obtained using the heuristic method, albeit the VEnCode quality was significantly increased for all estimates, as compared to the sampling method, as expected (*P <* 0.01; Tukey HSD post hoc test; Fig. 10b and c). We conclude that RE activity profiles obtained from single-cell sequencing data can be successfully integrated into the VEnCode pipeline when pooled estimates are used. At least for C1 CAGE sequencing of a small number of A549 cells, these estimates are not as optimal as those from bulk-sequencing data, due to their proneness to false negative RE calls and the generally reduced quality of the VEnCodes generated.

## Discussion

A major challenge in biomedicine is to access and gain control of a specific cellular type, be it in a healthy or disease state, within a complex and highly adaptable body. A methodology that allows genetic access to all cellular types and states in the human body would have a major impact in multiple domains of life science, including the possibility of studying and designing novel research tools and therapies, as well as better bioinspired technology and cosmetics. Such methodology addresses a major problem in the fields of life sciences research, biological engineering, and gene therapy: cellular-targeting, or how to restrict the desired genetic intervention to a unique set of cells within an organism or different cell states within unicellular populations.

Even when specific solutions exist (e.g., antibodies against target cell surface proteins or viruses with tropism towards certain cell types) that give access to a single cell type or state in an organism, no approach is known that allows for the systematic generation of similar specific solutions for other cell types or states in any given organism. Therefore, there is a profound limitation in the technologies available to genetically access particular cellular types and states in a very limited set of organisms.

An alternative to these procedures is to use methods that do not rely on cell-specific strategies to deliver genetic materials to cells: for instance, to use a system that delivers the desired genetic material to as many cells as possible in a complex organism, unrestrictedly. Considering that such systems are becoming available (e.g., unbiased non-integrating viral delivery or chemical-based delivery), the challenge becomes to have a highly versatile approach to activate any particular genetic message exclusively within a target cell type.

Intersectional genetics provides a solution for cellular targeting in complex organisms. However, there are several challenges that have to be overcome in order to apply intersectional genetics in human. The first challenge is the understandable lack of a library of gene drivers with known expression patterns to choose intersections from. To overcome this, we attempted to explore alternative resources, such as large RE usage databases determined using next-generation sequencing methods. We explored a curated panel of 154 primary human cell types and 158 cancer cell types for which a uniform RE usage atlas consisting of CAGE-seq data is currently available [3, 4, 29]. FANTOM5 data and other data sets have been previously explored as potential sources of cell-specific features, including enhancers [53, 54]. One of these tools is SlideBase, which uses interacting sliders for the selection of expressed features from a given data set by user-customized expression thresholds [53]. However, while such user-friendly tools can serve this and many other purposes, they are neither conceived nor optimized for a systematic analysis of intersectional genetics. An additional limitation is that the data sets have not been curated with the conservative criteria for unique cell types that we used.

The second challenge is to understand the landscape of intersectional genetics in human, including its safety and reliability. To the best of our knowledge, a systematic assessment of the potential and robustness of the intersectional genetics approach had never been performed for any organism, much less for humans. How far could intersectional genetics take us as an approach to gain accessibility to any given human cell? If each cell type does not have a uniquely active RE, or if the usage of a single unique RE carries high risks for therapeutic and diagnostic purposes due to technical artifacts and biological noise, would the unique intersection of 2 or more REs be enough to generate cell type–specific gene drivers for every human cell type? This is a relevant question, as there are several technical solutions available to explore genetically the intersections of active REs, such as split-transcription factors and recombinase-based strategies [14, 18–28].

We found herein that >90% of the primary human cell types surveyed can be safely and robustly distinguished from each other with as little as 3 to 4 REs. We called these combinations VEnCodes. VEnCodes can be defined as the smallest gene expression ON/OFF signature that carries enough diagnostic value to distinguish between the target cell and other non-target cell types within a complex mixture of cells. Clearly, VEnCodes with 1 and 2 REs exist and their technical exploitation is already feasible with current techniques. However, for many cells, more REs are required, either to obtain a VEnCode or to obtain a safer and more robust VEnCode. Hence, new intersectional methods are desirable to capitalize on the intersection of 3 or more active REs.

While we obtained VEnCodes for most cells using heuristic methods, we failed to obtain VEnCodes for ∼10% of the primary cells surveyed, even when 10 RE intersections were allowed. It is important to notice that we have by no means saturated the VEnCode search space. Hence, more thorough, brute-force methods (e.g., an exhaustive sampling method) might find VEnCodes for these difficult cell types. However, some of these cell types might indeed have poorly distinguishable or indistinguishable RE activity profiles. These cell types might require other techniques for detection. A possibility that was not explored here is to use other intersectional methods based on other Boolean logical operations, such as “OR," “NOT," and “NOR."

To create a quality index for VEnCodes, we determined its susceptibility to technical artifacts and biological noise using Monte Carlo simulations. We show that the average VEnCode quality varies significantly between different primary cell types, so that certain cells, such as mast cells and hepatocytes, are more safely distinguishable from others than most fibroblasts subtypes. VEnCode quality ranking could be further optimized by considering the original non-binarized RE activity profiles (TPMs). In this case, VEnCode quality would also depend on the RE with the lowest TPM, as this would be the limiting factor for each intersection.

By exploring RE usage data from the same primary cell type obtained from multiple donors, we find that VEnCodes are very (∼100%) robust, especially when determined using enhancer data. Promoter data-based VEnCodes for primary cell types increase when data from at least 2 cell type donors are used. It is not clear if this reduced robustness using single donor promoter data is due a technical or biological source of noise.

To probe the RE space in a cell state paradigm, we explored data from different cancer cell lines isolated from patients diagnosed with tumors of the same cellular origin. Several cancer cell types are hypervariable in nature, posing a challenge for finding a specific VEnCode that detects the same cancer cell type across multiple individuals. However, VEnCodes could be determined for all cancer types surveyed where multiple cell lines were available, even for notoriously hypervariable cancer cell types such as SCLC cells. Furthermore, as VEnCode retrieval can be systematized, in the absence of a single VEnCode that satisfies detection criteria for multiple cancer cell subtypes, multiple VEnCodes can be designed to account for cancer cell heterogeneity.

Finally, we showed that VEnCodes obtained from CAGE-seq data can be cross-validated using promoter and enhancer usage data obtained by other methods. In general, the extent of cross-validation varied greatly between cell types, a fact that can be partially explained by the imperfect correlation between CAGE-seq and the RE activity endpoints measured in the other databases. Intuitively, the methods of choice to cross-validate VEnCodes in target cells are methods that infer an enhancer function, such as simple luciferase assays or STARR-seq, where functional enhancers transcribe themselves [55]. Cross-validation of a second key assumption of VEnCodes—that they only function in the desired cell type and not in other cell types—is much more challenging. We partially addressed this challenge using a curated ENCODE DNAse-seq primary cell database, achieving significant cross-validation for CAGE-seq VEnCode specificity. Ultimately, the method of choice for CAGE-seq VEnCode cross-validation would be functional enhancer assays using a panel of complex human organoids.

VEnCodes can now be explored as minimal RE program-sensing parts that can be encoded genetically into plasmid-based biosensors, packaged into viral or non-viral systems, and delivered to cells in the body to diagnose whether or not the RE program of a given cell matches that of the VEnCode. Engineering biosensors that sense the activity of 3 to 4 REs and then perform a multiple “AND" gate computation to generate a single output is technically feasible with synthetic biology. Such genetic biosensors could revolutionize medicine by allowing safe and specific gene delivery to any cell type or cell state in the human body.

Enhancer-based VEnCodes are clearly the most promising combinations for generating intersectional genetics tools. Each enhancer can, for instance, be placed directly upstream of a general or synthetic basal promoter. In contrast, one needs to consider that promoter-based VEnCodes, such as those obtained in the heuristic2 method, might not necessarily autonomously convey the desired cell type–specific transcription when placed in a synthetic construct context. Nevertheless, there are many efforts to map enhancer-promoter interactions [3, 56, 57], which could be used to optimize the heuristic2 method.

Finally, we have shown that single cell–based strategies for RE estimation, such as C1 CAGE, are compatible with the VEnCode pipeline. The VEnCodes obtained were understandably of lower quality than those derived from bulk CAGE-seq data alone. Pooling data from larger numbers of cells can theoretically improve the VEnCode quality, considering larger numbers of REs are obtained. The low-confidence negative RE activity calls generated by pooled single cell–sequencing data are another point of concern, which might also be mitigated by pooling larger numbers of cells. Nevertheless, a major benefit of single-cell strategies for VEnCode applications is their potential to significantly increase the number of cell types with RE activity profiles. This potential to molecularly profile cell types and even to discover new cell types has become evident with single-cell RNA-seq [5–8]. Expanding the number of cell types in the curated single-cell RE activity database is critical, because it helps reduce the amount of false VEnCodes for all cell types (e.g., the larger the amount of cell types with robust known active RE calls, the less likely it is to obtain a VEnCodes that will fail in practice). Hence, the inclusion of a new cell type with a single cell–derived, partial RE profile based exclusively on sparse active RE calls is better than not having the profile at all. Finally, it would be interesting to verify in future studies whether, apart from integrating single-cell and bulk RE activity profiles, the algorithms and strategies described herein could also be applied to RE activity data generated exclusively from single-cell data.

## Conclusion

In summary, our results suggest that VEnCodes for a wide variety of human primary cell types and cancer cells can be discovered, quality-controlled, and cross-validated *in silico* using heuristic algorithms and publicly available genome-wide RE-usage databases, such as the FANTOM5 promoter and enhancer atlases. VEnCodes could be used to engineer intracellular biosensors or devices that use intersectional genetics tools to “read” the VEnCodes and translate them into a custom genetic output. This would allow systematic genetic access to any of these cell types or states. Genetic access carries enormous therapeutic potential by allowing the selective delivery of genetic messages and cures to cells, such as various forms of gene therapy or the specific genetic ablation of abnormal cancerous cells.

## Availability of source code and requirements

Project name: VEnCode

Project home page: https://github.com/AndreMacedo88/VEnCode

Operating system(s): Platform independent

Programming language: Python 3.6

Other requirements: None

License: BSD-3-Clause

RRID: SCR_018024

biotoolsID: https://bio.tools/VEnCode

CodeOcean: https://doi.org/10.24433/CO.3786894.v2

## Availability of supporting data and materials

Materials (code and data) are available at a github repository [58] and via the GigaScience database, GigaDB [59]. Python language [58] was used to implement all the algorithms and methods in this study (Python Software Foundation). Python Language Reference, version 3.6.5., is available online [60]. The R language with the ggplot2 package was used to generate the plots for the Figures [61, 62]. A computational capsule to provide examples of basic usage of the VEnCode package and to run the package's tests in a reproducible way is available via CodeOcean [63].

## Abbreviations

ANOVA: analysis of variance; CAGE-seq: capped analyses of gene expression sequencing; hiPSC: human-induced pluripotent stem cell; RE: regulatory element; SCLC: small cell lung carcinoma; TPM: tags per million; VEnCodes: versatile entry codes.

## Competing interests

The authors declare no conflicts of interests.

## Funding

A.M.G. was supported by an FCT (Fundação para a Ciência e Tecnologia) Investigator Grant (IF/00022/2012). A.M. was supported by fellowships from FCT (SFRH/BD/94931/2013) and the Liga Portuguesa Contra o Cancro (LPCC 2017). Work in the Integrative Biomedicine Laboratory is supported by the FCT (UID/Multi/04462/2019; PTDC/BEXBCM/1370/2014; PTDC/MED-NEU/30753/2017; and PTDC/BIA-BID/31071/2017), the MIT Portugal Program (MIT-EXPL/BIO/0097/2017), and FAPESP (Fundação de Amparo à Pesquisa do Estado de São Paulo) (2016/09659-3).

## Authors' contributions

A.M. and A.M.G. designed the study, implemented analyses, analyzed the data, and wrote the manuscript.

## Supplementary Material

giaa083_GIGA-D-19-00415_Original_Submission

giaa083_GIGA-D-19-00415_Revision_1

giaa083_Response_to_Reviewer_Comments_Original_Submission

giaa083_Reviewer_1_Report_Original_Submission Rani Elkon -- 1/14/2020 Reviewed

giaa083_Reviewer_1_Report_Revision_1 Rani Elkon -- 6/9/2020 Reviewed

giaa083_Reviewer_2_Report_Original_Submission Rory Kirchner -- 2/4/2020 Reviewed

giaa083_Reviewer_2_Report_Revision_1 Rory Kirchner -- 4/8/2020 Reviewed

giaa083_Supplemental_File

## Supplementary Data

Supplementary materials include Supplementary Figures S1–6, Supplementary Tables S1-S7, and Supplementary Data S1–2. The latter are available online [60].
