# Supplementary material for: The intersectional genetics landscape for humans
Source: Gigascience. 2020 Aug 6;9(8):giaa083. doi: 10.1093/gigascience/giaa083 (PMC7407247; doi:10.1093/gigascience/giaa083)
Supplement: giaa083_Response_to_Reviewer_Comments_Original_Submission [file giaa083_response_to_reviewer_comments_original_submission.pdf]

Author's Response To Reviewer Comments

Close

Point-by-point response to reviewer’s comments

Reviewer #1
* (Remarks to the Author): In this study Macedo and Gontijo systematically explore the potential of intersectional genetics to specifically target a particular cell type on the background of a large set of non-target cells. The study builds on FANTOM5 CAGE data that quantify promoter and enhancer activity, and presents different methods - based on heuristics and random sampling - for finding sets of k active regulatory elements (REs) whose intersection is specifically ON in the selected target cell type.
Interestingly, the analysis finds that >90% of the 154 analyzed cell types can be specifically targeted by an intersection of only 3-4 REs. Such intersections of REs that are specifically active in a selected target cell type, called by the authors - "Versatile Entry Codes" (VEnCodes), hold promise for advancing gene delivery approaches.
Overall, this is a nice work that examines a timely research question. It is well written and discuss different limitations of the analyses that were carried out.

R: We thank Reviewer #1 for the encouraging and constructive words and for the important comments that have helped us improve the manuscript.

Major comment:
* I find that the VEnCode cross-validation section (Figure 9) is the weaker part of the study, both conceptually and in terms of the results achieved. Conceptually, the validation that was carried out only examined the fraction of REs per VEnCode's that were found active in the respective cell type in an external DB. This does not consider at all specificity of targeting which is the essential aspect here. In addition to FANTOM5, ENCODE and RoadMap Epigenomics provide too extensive datasets on RE activity (using DHS). These resources can be used for cross validation. Indeed, the different techniques used (CAGE vs DHS) make this cross validation challenging. Yet, if the VEnCodes detected on this study are reliable some non-random agreement should be observed between these FANTOM5 and these external datasets. Furthermore, large expression data resources could be used for cross validation as well (considering gene expression level of a gene as a proxy for its promoter activity).
This way, expression data from TCGA can be used to cross validate (promoter) VEnCodes that were found on cancer cell types. GTEx is another gene expression resource that may be useful for such validation.

R: We agree with Reviewer #1 that cross-validation for specificity is an important aspect, and also agree that it is a challenging cross-validation to perform due to the different characteristics of the techniques applied to infer RE activity. This imperfect correlation could lead to technical noise unrelated to biological aspects. As we expect this noise to be even higher if we used non-RE data as mRNA levels for cross-validation, because the computational associations between transcript and specific REs are themselves imperfect correlations and mostly reflect inferences that have not been proven experimentally, we chose to follow one of the recommendations from the Reviewer #1 and use data obtained from techniques that mapped “REs” directly, such as DNAse-seq.

We thus obtained a large dataset of DNAse Hypersensitive Sites (DHS) from the ENCODE project, which is a surrogate for REs. We curated the dataset for single cell types matching the CAGE-seq database, and asked if there would be a non-random agreement between the VEnCode predictions based on FANTOM5 CAGE-Seq data and the ENCODE DHS profiles for the same cells. We find that there is indeed a non-random association between the predictions. We have added the required text to the Results and Discussion sections, and added panels E and F to Figure 9 and Supplementary Figure S9. All modified text is highlighted in blue in the main manuscript file.

Minor comment:
* ~10%-15% of the cells cannot be distinguished by the applied methodology. Could the authors present a hierarchical clustering for the analyzed samples and mark on the dendrogram those for which

a VEnCode was not found? I would expect those samples to be grouped into tight branches (and thus the difficulty to spate each one from the others).

R: We agree with Reviewer #1 and have included a hierarchical clustering of the analyzed samples and have marked the cells for which no VEnCode was found as a function of k RE elements. There are indeed some clusters of more difficult cell types, e.g., fibroblasts and white blood cells, which require more than k = 4 to generate a VEnCode or that we cannot find VEnCodes at all even with k = 10.
To address this point, we have added the required text to the results section, and added Supplementary Figures S2 and S3. All modified text is highlighted in blue in the main manuscript file.

* The quality of a VEnCode was evaluated by its robustness. An additional criterion is the strength of the RE activity in the target cell type. Quantitative information is not taken into account by the presented computational strategies (after setting cutoffs, the data is binary). I think it could be relevant to discuss this point in Discussion.

R: We agree with Reviewer #1 and have included in the discussion section the following text on this important point:
"VEnCode quality ranking could be further optimized by considering the original non-binarized RE activity profiles (TPMs). In this case, VEnCode quality would also depend on the RE with the lowest TPM, as this would be the limiting factor for each intersection."
All modified text is highlighted in blue in the main manuscript file.

Reviewer #2:
* (Remarks to the Author): I encountered a not identical but very similar problem this paper solves a few months ago while trying to find sets of markers that minimized the amount of stains we needed to do to distinguish dozens of cell types using non-specific markers. There wasn't really anything out there to do it. When I was looking around for solutions I found the math problem that this describes and the problem was NP-hard, but I forget the name of the problem. I realize this is incredibly useless, but there is a name for this general problem, it would be nice to reference it if you can figure out what it is. If I remember or find it again after sending this review, I'll send it along. I ended up writing something to solve my problem, but I had a simpler problem to deal with where I had a set of 20-30 markers and they wanted to find a minimal set of those that could uniquely identify the cells (https://github.com/roryk/mastermind), so I could solve the problem by deleting markers until I ended up with the smallest set. Your heuristic method is awesome and it is great that it scales to a lot more cell types and a lot more markers. Your idea of perturbing the codes to test for robustness is also awesome. I pointed my collaborator at your https://github.com/AndreMacedo88/VEnCode repository and your preprint and I put a note on my repository to look at yours for a much better solution.

R: We thank Rory Kirchner for the very encouraging and constructive words and for all the important comments that have helped us improve the manuscript. Thank you also for sharing the https://github.com/roryk/mastermind software. It was very informative and instructive.

About the complexity problem, we could define finding a VEnCode as a decision problem with an algorithm of polynomial time complexity O((c*r)^k). Unfortunately, we could not characterize the name of the problem further with confidence without further work even after consulting with a mathematician and computational scientist. We added the description of the polynomial time complexity when describing the algorithm in the Results section.

* Regarding your choice of license, it's your call and definitely should not be considered regarding your paper's status, but I think the academic-only licenses kind of stink. It is unlikely you're going to make any money from it and you'll end up having a lot less uptake and use of your awesome software. https://liorpachter.wordpress.com/2017/08/03/i-was-wrong-part-2/ has a nice discussion of the downsides of having an academic-only license. Kudos for including a nice code of conduct.

R: Thank you for pointing out Lior Pachter's discussion and the rationale behind this choice. We agree and have accordingly chosen a permissive license, the BSD-3-Clause License.

* I had a few problems getting VEnCode installed, but I fixed them and opened up a pull request which I am sure will be merged in. The tests don't seem to work, and there isn't any example data included with which we can run the tool, which would be a nice and looks like it was intended. It would also be nice if there was some CI set up along with functional tests so we could have a good example of how to run the

tool, if that is something possible to do with a mini-dataset.

R: Thank you very much for testing the software. We apologize for the installation problems. We have fixed it and have included the mini-dataset for running the tests. We also included a tutorial on how to run the tests, as well as examples on how to use the tool in both GitHub (https://github.com/AndreMacedo88/VEnCode) and the CodeOcean (https://codeocean.com/capsule/7611480/tree/v2) pages for the tool. Finally, installation for users is now much easier since the tool is now uploaded to PyPi and available to install by Pip.

* This paper should definitely be accepted, I will be using this software in the future for sure. I clicked Minor Revision just because it would be great to have an example dataset that we could run and make sure the tool is actually working. That is the only thing I care about regarding a revision. The idea and the paper are great.
Best,
Rory Kirchner (roryk@alum.mit.edu)

R: Thank you very much. This was extremely stimulating for us and we are very happy that the software is helpful.

Close
