# Supplementary material for: The intersectional genetics landscape for humans
Source: Gigascience. 2020 Aug 6;9(8):giaa083. doi: 10.1093/gigascience/giaa083 (PMC7407247; doi:10.1093/gigascience/giaa083)
Supplement: giaa083_Reviewer_1_Report_Original_Submission — Rani Elkon -- 1/14/2020 Reviewed [file giaa083_reviewer_1_report_original_submission.pdf]

Reviewer Report

Title: The intersectional genetics landscape for human

Version: Original Submission **Date:** 1/14/2020

Reviewer name: Rani Elkon

Reviewer Comments to Author:

In this study Macedo and Gontijo systematically explore the potential of intersectional genetics to specifically target a particular cell type on the background of a large set of non-target cells. The study builds on FANTOM5 CAGE data that quantify promoter and enhancer activity, and presents different methods - based on heuristics and random sampling - for finding sets of k active regulatory elements (REs) whose intersection is specifically ON in the selected target cell type. Interestingly, the analysis finds that >90% of the 154 analyzed cell types can be specifically targeted by an intersection of only 3-4 REs. Such intersections of REs that are specifically active in a selected target cell type, called by the authors - "Versatile Entry Codes" (VEnCodes), hold promise for advancing gene delivery approaches. Overall, this is a nice work that examines a timely research question. It is well written and discuss different limitations of the analyses that were carried out.

Major comment:

I find that the VEnCode cross-validation section (Figure 9) is the weaker part of the study, both conceptually and in terms of the results achieved. Conceptually, the validation that was carried out only examined the fraction of REs per VEnCode's that were found active in the respective cell type in an external DB. This does not consider at all specificity of targeting which is the essential aspect here. In addition to FANTOM5, ENCODE and RoadMap Epigenomics provide too extensive datasets on RE activity (using DHS). These resources can be used for cross validation. Indeed, the different techniques used (CAGE vs DHS) make this cross validation challenging. Yet, if the VEnCodes detected on this study are reliable some non-random agreement should be observed between these FANTOM5 and these external datasets. Furthermore, large expression data resources could be used for cross validation as well (considering gene expression level of a gene as a proxy for its promoter activity). This way, expression data from TCGA can be used to cross validate (promoter) VEnCodes that were found on cancer cell types. GTEx is another gene expression resource that may be useful for such validation.

Minor comment:

* ~10%-15% of the cells cannot be distinguished by the applied methodology. Could the authors present a hierarchical clustering for the analyzed samples and mark on the dendrogram those for which a VEnCode was not found? I would expect those samples to be grouped into tight branches (and thus the difficulty to spate each one from the others).

* The quality of a VEnCode was evaluated by its robustness. An additional criterion is the strength of the RE activity in the target cell type. Quantitative information is not taken into account by the presented computational strategies (after setting cutoffs, the data is binary). I think it could be relevant to discuss this point in Discussion.

Methods

Are the methods appropriate to the aims of the study, are they well described, and are necessary controls included? Choose an item.

Conclusions

Are the conclusions adequately supported by the data shown? Choose an item.

Reporting Standards

Does the manuscript adhere to the journal's guidelines on minimum standards of reporting? Choose an item.

Choose an item.

Statistics

Are you able to assess all statistics in the manuscript, including the appropriateness of statistical tests used? Choose an item.

Quality of Written English

Please indicate the quality of language in the manuscript: Choose an item.

Declaration of Competing Interests

Please complete a declaration of competing interests, considering the following questions:

- Have you in the past five years received reimbursements, fees, funding, or salary from an organisation that may in any way gain or lose financially from the publication of this manuscript, either now or in the future?
- Do you hold any stocks or shares in an organisation that may in any way gain or lose financially from the publication of this manuscript, either now or in the future?
- Do you hold or are you currently applying for any patents relating to the content of the manuscript?
- Have you received reimbursements, fees, funding, or salary from an organization that holds or has applied for patents relating to the content of the manuscript?
- Do you have any other financial competing interests?
- Do you have any non-financial competing interests in relation to this paper?

If you can answer no to all of the above, write 'I declare that I have no competing interests' below. If your reply is yes to any, please give details below.

I declare that I have no competing interests

I agree to the open peer review policy of the journal. I understand that my name will be included on my report to the authors and, if the manuscript is accepted for publication, my named report including any attachments I upload will be posted on the website along with the authors' responses. I agree for my report to be made available under an Open Access Creative Commons CC-BY license (http://creativecommons.org/licenses/by/4.0/). I understand that any comments which I do not wish to be included in my named report can be included as confidential comments to the editors, which will not be published.

Choose an item.

To further support our reviewers, we have joined with Publons, where you can gain additional credit to further highlight your hard work (see: https://publons.com/journal/530/gigascience). On publication of this paper, your review will be automatically added to Publons, you can then choose whether or not to claim your Publons credit. I understand this statement.

Yes Choose an item.
