# Supplementary material for: The intersectional genetics landscape for humans
Source: Gigascience. 2020 Aug 6;9(8):giaa083. doi: 10.1093/gigascience/giaa083 (PMC7407247; doi:10.1093/gigascience/giaa083)
Supplement: giaa083_Reviewer_2_Report_Original_Submission — Rory Kirchner -- 2/4/2020 Reviewed [file giaa083_reviewer_2_report_original_submission.pdf]

Reviewer Report

Title: The intersectional genetics landscape for human

Version: Original Submission **Date:** 2/4/2020

Reviewer name: Rory Kirchner

Reviewer Comments to Author:

I encountered a not identical but very similar problem this paper solves a few months ago while trying to find sets of markers that minimized the amount of stains we needed to do to distinguish dozens of cell types using non-specific markers. There wasn't really anything out there to do it. When I was looking around for solutions I found the math problem that this describes and the problem was NP-hard, but I forget the name of the problem. I realize this is incredibly useless, but there is a name for this general problem, it would be nice to reference it if you can figure out what it is. If I remember or find it again after sending this review, I'll send it along. I ended up writing something to solve my problem, but I had a simpler problem to deal with where I had a set of 20-30 markers and they wanted to find a minimal set of those that could uniquely identify the cells (https://github.com/roryk/mastermind), so I could solve the problem by deleting markers until I ended up with the smallest set. Your heuristic method is awesome and it is great that it scales to a lot more cell types and a lot more markers. Your idea of perturbing the codes to test for robustness is also awesome. I pointed my collaborator at your https://github.com/AndreMacedo88/VEnCode repository and your preprint and I put a note on my repository to look at yours for a much better solution.

Regarding your choice of license, it's your call and definitely should not be considered regarding your paper's status, but I think the academic-only licenses kind of stink. It is unlikely you're going to make any money from it and you'll end up having a lot less uptake and use of your awesome software. https://liorpachter.wordpress.com/2017/08/03/i-was-wrong-part-2/ has a nice discussion of the downsides of having an academic-only license. Kudos for including a nice code of conduct.

I had a few problems getting VEnCode installed, but I fixed them and opened up a pull request which I am sure will be merged in. The tests don't seem to work, and there isn't any example data included with which we can run the tool, which would be a nice and looks like it was intended. It would also be nice if there was some CI set up along with functional tests so we could have a good example of how to run the tool, if that is something possible to do with a mini-dataset.

This paper should definitely be accepted, I will be using this software in the future for sure. I clicked Minor Revision just because it would be great to have an example dataset that we could run and make sure the tool is actually working. That is the only thing I care about regarding a revision. The idea and the paper are great.

Best,

Rory Kirchner (roryk@alum.mit.edu)

Methods

Are the methods appropriate to the aims of the study, are they well described, and are necessary controls included? Choose an item.

Conclusions

Are the conclusions adequately supported by the data shown? Choose an item.

Reporting Standards

Does the manuscript adhere to the journal's guidelines on minimum standards of reporting? Choose an item.

Choose an item.

Statistics

Are you able to assess all statistics in the manuscript, including the appropriateness of statistical tests used? Choose an item.

Quality of Written English

Please indicate the quality of language in the manuscript: Choose an item.

Declaration of Competing Interests

Please complete a declaration of competing interests, considering the following questions:

- Have you in the past five years received reimbursements, fees, funding, or salary from an organisation that may in any way gain or lose financially from the publication of this manuscript, either now or in the future?
- Do you hold any stocks or shares in an organisation that may in any way gain or lose financially from the publication of this manuscript, either now or in the future?
- Do you hold or are you currently applying for any patents relating to the content of the manuscript?
- Have you received reimbursements, fees, funding, or salary from an organization that holds or has applied for patents relating to the content of the manuscript?
- Do you have any other financial competing interests?
- Do you have any non-financial competing interests in relation to this paper?

If you can answer no to all of the above, write 'I declare that I have no competing interests' below. If your reply is yes to any, please give details below.

I declare that I have no competing interests

I agree to the open peer review policy of the journal. I understand that my name will be included on my report to the authors and, if the manuscript is accepted for publication, my named report including any attachments I upload will be posted on the website along with the authors' responses. I agree for my report to be made available under an Open Access Creative Commons CC-BY license (http://creativecommons.org/licenses/by/4.0/). I understand that any comments which I do not wish to be included in my named report can be included as confidential comments to the editors, which will not be published.

Choose an item.

To further support our reviewers, we have joined with Publons, where you can gain additional credit to further highlight your hard work (see: https://publons.com/journal/530/gigascience). On publication of this paper, your review will be automatically added to Publons, you can then choose whether or not to claim your Publons credit. I understand this statement.

Yes Choose an item.
